# Hospital control and multidrug-resistant pulmonary tuberculosis in female patients, Lima, Peru.

**DOI:** 10.3201/eid0701.010117

**Published:** 2001

**Authors:** F F Willingham, T L Schmitz, M Contreras, S E Kalangi, A M Vivar, L Caviedes, E Schiantarelli, P M Neumann, C Bern, R H Gilman, Working Group on TB in Peru

**Affiliations:** University of Maryland, School of Medicine, Baltimore, Maryland, USA.

## Abstract

We examined the prevalence of tuberculosis (TB), rate of multidrug-resistant (MDR) TB, and characteristics of TB on a female general medicine ward in Peru. Of 250 patients, 40 (16%) were positive by sputum culture and 27 (11%) by smear, and 8 (3%) had MDRTB. Thirteen (33%) of 40 culture-positive patients had not been suspected of having TB on admission. Six (46%) of 13 patients whose TB was unsuspected on admission had MDRTB, compared with 2 (7%) of 27 suspected cases (p = 0.009). Five (63%) of 8 MDRTB patients were smear positive and therefore highly infective. In developing countries, hospital control, a simple method of reducing the spread of MDRTB, is neglected.


### Hospital Control and Multidrug-Resistant Pulmonary Tuberculosis in Female Patients, Lima, Peru

**Field F. Willingham*†‡ Tracy L. Schmitz,‡§ Macbeth Contreras,‡ Sheela E. Kalangi,‡¶ Aldo M. Vivar,‡ Luz Caviedes,# Eduardo Schiantarelli,** Paola Maurtua Neumann,†‡ Caryn Bern,‡‡ Robert H. Gilman,†‡# and the Working Group on TB in Peru[1]**

*University of Maryland, School of Medicine, Baltimore, Maryland, USA; †Johns Hopkins University School of Hygiene and Public Health, Baltimore, Maryland, USA; ‡Proyectos en Informática, Salud, Medicina, y Agricultura (PRISMA), Lima, Peru; §Tufts University School of Medicine, Boston, Massachusetts, USA; ¶University of Arizona School of Public Health, Phoenix, Arizona, USA; #Universidad Peruana Cayetano Heredia, Lima, Peru; **Arzobispo Loayza Hospital, Lima, Peru; ‡‡Centers for Disease Control and Prevention, Atlanta, Georgia, USA.

We examined the prevalence of tuberculosis (TB), rate of multidrug-resistant (MDR) TB, and characteristics of TB on a female general medicine ward in Peru. Of 250 patients, 40 (16%) were positive by sputum culture and 27 (11%) by smear, and 8 (3%) had MDRTB. Thirteen (33%) of 40 culture-positive patients had not been suspected of having TB on admission. Six (46%) of 13 patients whose TB was unsuspected on admission had MDRTB, compared with 2 (7%) of 27 suspected cases (p=0.009). Five (63%) of 8 MDRTB patients were smear positive and therefore highly infective. In developing countries, hospital control, a simple method of reducing the spread of MDRTB, is neglected.

From 1990 to 2000, tuberculosis (TB) caused an estimated 88 million new infections and 30 million deaths worldwide (1). In Peru, tuberculosis is highly endemic; a shantytown in Lima had an annual incidence of pulmonary tuberculosis of 364 per 100,000 population (2). Despite the implementation of community-based treatment and control programs in Peru (3), management of the disease has been complicated by high rates of multidrug-resistant (MDR) TB. In one study in Peru, 4.5% of all reported cases were resistant to isoniazid and rifampin (4). Nosocomial spread of MDRTB has been reported in both industrialized and developing countries and has been linked to inadequate hospital infection control practices (5-7).

Address for correspondence: Robert H. Gilman, Department of International Health, Johns Hopkins University School of Hygiene and Public Health, 615 North Wolfe St., Baltimore, MD 21205, USA; fax: 410-550-6733; e-mail: rgilman@jhsph.edu.

We investigated the potential for nosocomial spread of MDRTB in one city hospital in Lima. We assessed the prevalence of TB among hospitalized patients on a general medicine ward, the rate of MDRTB and the extent to which active pulmonary TB had been suspected in patients at the time of admission.

### Methods

### Study Population and Design

The study was conducted from January to December 1997 in the Arzobispo Loayza Hospital, an urban public hospital in Lima, Peru. This hospital was founded as a women's hospital in the eighteenth century and continues to serve

[1]Other members of the Working Group on TB in Peru include Oswaldo Bisbal, Anna Bowen, Rosa Cama, William Checkley, Scott Franzblau, Luis Miguel Frachie, Patricia Fuentes, Hugo Garcia, Guillermo Lescano, Cheryl Liechty, Sonia Montenegro, Guillermo Salazar, Patricia Sheen, Eduardo Ticona, Teresa Valencia, and Richard Witzig.

a largely female patient population. We solicited the participation of all patients admitted to one of the hospital's eight female internal medicine wards (an open room with 30 beds) during the study period. The most common admission diagnoses over the year of study were pneumonia, bronchiectasis, cardiac insufficiency, TB, cellulitis, diabetes mellitus and chronic renal failure. The study protocol was approved by the institutional review boards of the Johns Hopkins University and Loayza Hospital. All study participants gave informed consent.

Patients who agreed to participate in the study answered a brief questionnaire and underwent physical examination. The medical records were reviewed. A tuberculin skin test (TST) (5 tuberculin units, Connaught, Swiftwater, PA) was administered and was read after 48 to 72 hours. The TST was considered positive if the area of induration measured $\geq$10 mm both vertically and horizontally. At least one sputum specimen >1 mL in volume was obtained; whenever possible, additional sputum specimens were obtained on consecutive days.

### Laboratory Testing for TB

#### Acid-fast Bacilli Smear Microscopy

All samples were digested and concentrated by the standard N-acetyl-L-cysteine NaOH-Na citrate method for processing mycobacterial specimens (8). Ziehl-Neelsen and Auramine staining were performed by standard techniques (8).

#### Cultures

Mycobacterial growth indicator tubes (Becton Dickinson, Sparks, MD) containing both 10% OADC (oleic acid, albumin, dextrose, and catalase) (Becton Dickinson, Sparks, MD), and 100 µL of PANTA Antimicrobic Supplement (Polymyxin B, Amphotericin B, Nalidixic acid, Trimethoprim, and Azlocillin) (Becton Dickinson) were injected with 500 µL of decontaminated sputum sample according to the manufacturer's specifications. Löwenstein-Jensen slants (Difco, Detroit, MI) and Middlebrook 7H11 medium plates (Difco, Detroit, MI) were injected with 250 µL of decontaminated sample. Tubes were incubated at 37°C and examined for mycobacterial growth at least weekly for up to 6 weeks with a 365-nm UV transilluminator. Löwenstein-Jensen slants and micro-agar 7H11 plates were incubated at 37°C with and without 5% $CO_2$ and examined by light microscopy for mycobacterial growth at least weekly for 2 to 8 weeks after injection (8). Criteria for positive mycobacterial growth have been previously described by the Centers for Disease Control (9).

#### Sensitivity Testing

The microplate alamar blue assay was used to determine mycobacterial drug resistance (10). Bacterial suspensions were prepared from colonies grown on Middlebrook 7H11 agar. Samples of the bacterial suspension (20 µL) were grown in 96-well plates containing serial dilutions of anti-TB drugs (isoniazid, rifampin, ethambutol, streptomycin, capreomycin, ciprofloxacin) until control wells tested positive for mycobacterial growth, usually in 5 to 6 days. Alamar blue reagent was then added to each well, and mycobacterial growth was identified by a change in media color from blue to pink. MIC was defined as the lowest drug concentration at which no blue-to-pink color change was observed. MICs for the panel of six anti-TB drugs were determined for each isolate.

### Data Analysis

Patients were included in the study if they completed the questionnaire, had a physical examination, and provided one adequate sputum specimen. A patient was considered to have MDRTB if the sputum exhibited growth in media containing both isoniazid and rifampin. HIV tests were not performed as part of this study, but HIV test results were available for some patients.

All data were entered twice, and the two databases were compared to eliminate data entry errors. Data were analyzed with SPSS version 7.5 (SPSS Inc., Chicago, IL) and Epi Info version 6.0 (CDC, Atlanta, GA). The chi-square and Fisher's exact tests were used to measure strengths of association for categorical variables. The Wilcoxon 2-sample test was used to compare continuous variables.

### Results

From January to December 1997, 250 (78%) of 319 patients admitted to the ward had a completed questionnaire and physical examination and at least one adequate sputum specimen. Forty patients (16%) had sputum cultures positive for *Mycobacterium tuberculosis*, and 26 of these had positive sputum smears. One patient had a positive smear but a negative culture. Only three patients had a diagnosis of HIV infection; none of the three had a positive sputum specimen. Of the 69 ward patients who declined to participate or were unable to provide an adequate sputum specimen, 4 (6%) had been admitted with a diagnosis of suspected TB. If we assume all excluded patients to be negative for TB, the minimum estimated TB prevalence on the ward was 13%.

Patients with a cough of any duration, a cough that lasted >2 weeks, reported weight loss, hemoptysis, or a family history of TB were more likely to have sputum cultures positive for TB (Table 1). Anorexia was associated with a lower likelihood of TB. Because of logistic constraints, we were able to place and read a TST at 48 to 72 hours only on a subset of patients. Of the 67 patients with TST results, a positive reading was observed in 11 (55%) of 20 culture-positive patients compared with 10 (21%) of 47 patients without TB (p=0.007). Among culture-positive patients, those with a positive TST response were younger than those with a negative reading (median 23 years of age[range 19-66] vs. 47 years [range 25-88], p=0.02 by Wilcoxon 2-sample test). The socioeconomic status of patients with and without TB was similar.

Table 1. Female patients admitted to a general medicine ward of a hospital, Lima, Peru

| Characteristic | *Mycobacterium tuberculosis* culture results | |
|---|---|---|
| | Positive N=40, n (%) | Negative[a] N=209, n (%) |
| Median age (range) | 43 (18-96) | 46 (14-92) |
| Cough | 35 (88)[b] | 125 (60)[b] |
| Cough for ≥ 2 weeks | 25 (63)[b] | 64 (31)[b] |
| Weight loss | 33 (83)[b] | 122 (58)[b] |
| Hemoptysis | 12 (30)[c] | 29 (14)[c] |
| Anorexia | 22 (55)[c] | 149 (71)[c] |
| Fever | 24 (60) | 108 (51) |
| Dyspnea | 22 (55) | 107 (51) |
| TST positive[d] | 11 (55)[c] | 10 (21)[c] |
| BCG scar | 28 (70) | 150 (71) |
| History of BCG vaccination | 29 (73) | 152 (72) |
| Family history of TB | 12 (30)[c] | 32 (15)[c] |
| Prior history of TB | 9 (23) | 34 (16) |
| Socioeconomic indicators | | |
| Electricity in home | 36 (90) | 196 (93) |
| Piped water | 32 (80) | 180 (86) |
| Able to read and write | 31 (78) | 159 (76) |

[a]One patient who was smear positive but culture negative was excluded from the analysis.
[b]P value < 0.01 by Mantel-Haenzsel chi-square test.
[c]P value < 0.05 by Mantel-Haenzsel chi-square test.
[d]A total of 67 patients, 20 *M. tuberculosis* culture-positive and 47 *M. tuberculosis* culture-negative, had tuberculin skin tests (TST).

Of the 181 patients who reported past BCG immunization, 178 (98%) had a scar. No vaccine scars were observed among the 68 persons who reported no history of BCG immunization. However, having a BCG scar was not associated with any apparent protective effect (Table 1). The presence of a BCG scar was not associated with a positive TST, even when TB culture positive patients were excluded (p=0.7).

Of 40 patients with at least one positive sputum culture, 23 (58%) had strains resistant to at least isoniazid, 8 (20%) to rifampin, 4 (10%) to ethambutol, and 1 (3%) to streptomycin. None were resistant to ciprofloxacin or capreomycin. Eight patients (20%) had TB resistant to both isoniazid and rifampin and were classified as having MDRTB. All 8 patients with resistance to rifampin also had resistance to isoniazid, and 15 patients had strains resistant to isoniazid but not to rifampin. Of the eight strains resistant to both isoniazid and rifampin, one was also resistant to ethambutol, one to streptomycin, and one to both ethambutol and streptomycin. Of 8 patients with MDRTB, 3 had a previous history of TB treatment.

Culture-positive patients for whom TB was the admitting diagnosis differed from those in whom TB was not suspected at the time of admission (Table 2). Patients whose TB had not been suspected were older and less likely to have the classic findings of cough, hemoptysis, weight loss, and prior personal or family history of TB. Patients whose TB had not been suspected at the time of admission were less likely to have a positive sputum smear, but this difference did not reach statistical significance (p=0.16 by Fisher's exact test). However, patients whose TB had not been suspected were significantly more likely to have MDRTB. Six (75%) of 8 patients with MDRTB were not suspected to have TB on admission; 3 (50%) of these six were also smear positive. Admitting diagnoses among culture-positive patients whose TB had not been suspected on admission included two patients with diabetes mellitus, one with systemic lupus erythematosus, and one with a lung lesion thought to be a hydatid cyst.

Table 2. *Mycobacterium tuberculosis* culture-positive patients, by admission diagnosis, Lima, Peru

| | *M. tuberculosis* culture-positive patients | |
|---|---|---|
| Characteristic | Suspected TB N=27, n (%) | No suspected TB N=13, n (%) |
| Median age (range) | 27 (18-87)[a] | 58 (22-96)[a] |
| Cough | 27 (100)[b] | 8 (62)[b] |
| Cough for ≥ 2 weeks | 20 (74)[c] | 5 (39)[c] |
| Weight loss | 25 (93)[c] | 8 (62)[c] |
| Hemoptysis | 10 (37) | 2 (15) |
| Fever | 16 (59) | 8 (62) |
| Anorexia | 13 (48) | 9 (69) |
| Dyspnea | 17 (63) | 5 (39) |
| Prior history of TB | 8 (30) | 1 (8) |
| Family history of TB | 10 (37) | 2 (15) |
| Smear positive | 20 (74) | 6 (46) |
| MDRTB | 2 (7)[c] | 6 (46)[c] |
| MDRTB and smear positive | 2 (7) | 3 (23) |

[a]p value $< 0.05$ by Wilcoxon 2-sample test.
[b]p value $< 0.01$ by Fisher's exact 2-tailed test.
[c]p value $< 0.05$ by Fisher's exact 2-tailed test.
MDRTB = Multidrug-resistant tuberculosis.

### Conclusions

The overall prevalence of TB among our study patients was high: at least 13% of all patients admitted to this general medicine ward had active TB. Two-thirds of TB patients were smear positive and therefore highly infectious, one-fifth had multidrug-resistant strains, and 75% of the patients with MDRTB had not been suspected of having TB when they entered the hospital. As in most Latin American hospitals, no masks or other respiratory devices were used to prevent spread in this hospital, even when the patient was known to be smear positive and highly infectious.

Nosocomial outbreaks of MDRTB in the United States in the 1980s and early 1990s heightened enforcement of stringent hospital control measures (11), leading to measurable decreases in TST conversion rates among hospital staff (12). Although the rate of TB in Peru is approximately 20 times higher than that of New York City (13), no concerted effort has been made to improve TB control measures in Peruvian hospitals.

The spread of MDRTB threatens control efforts (14). The fact that the majority of our patients with MDRTB had no history of past treatment of TB implies that person-to-person transmission of multidrug resistant strains occurs in Peru. Our data suggest that hospital wards may be one of the sites of transmission.

In developing countries where resources are limited, TB control programs focus on identification and treatment of infectious cases (15). Although treatment is clearly an important component of control, person-to-person spread of resistant strains makes isolation a high priority for preventing transmission. TST testing was not useful in identifying the group in need of screening. Anergy, which was common among culture-positive TB cases, was associated statistically with older median age and was perhaps related to concurrent systemic illness and poor nutritional status among hospitalized patients.

Although Peru has implemented an effective community-based TB control program, hospital control has not been a focus. Control measures such as isolation and respiratory precautions, stringently enforced in the past, were relaxed worldwide after the advent of inexpensive, effective anti-TB medications. After 50 years of selective drug pressure, the outbreak of MDRTB in New York City (5) dramatically highlighted the consequences of lapses in infection control.

Our data show that in countries or locales with a known high prevalence of TB, hospitals should screen all patients with respiratory symptoms by sputum smear within 12 hours of admission to hospital. Those found to be smear-positive should be placed in respiratory isolation, apart from TB-negative patients, until the smear becomes negative. Hospital personnel should observe respiratory precautions in caring for these patients. A system of rapid culture diagnosis and susceptibility testing should be implemented, allowing the presumptive diagnosis of MDRTB within 2 weeks (16). In combination, admission screening for TB, re-implementation of effective hospital respiratory control, and rapid TB diagnosis can substantially decrease the transmission of TB, especially MDRTB, in countries like Peru.

### Acknowledgments

We thank R. Black, D. Berg, and K. Laserson for helpful comments and J.B. Phu and D. Sara for their assistance.

This study was supported in part by NIH grant number U01-AI35894-03, World AIDS Federation grant number 94.093, Fogarty, FIRCA TW00611 and ITREID and the anonymous RG-ER fund.

Mr. Willingham, a fourth-year medical student at the University of Maryland, performed this study after he completed his Masters in Public Health at the Johns Hopkins School of Public Health. His research interests focus on tuberculosis, infectious diseases, and public health.

### References

1. Dolin PJ, Raviglione MC, Kochi A. Global tuberculosis incidence and mortality during 1990-2000. Bull World Health Organ 1994; 72:213-20.
2. Sanghavi DM, Gilman RH, Lescano-Guevara AG, Checkley W, Cabrera LZ, Cardenas V. Hyperendemic pulmonary tuberculosis in a Peruvian shantytown. Am J Epidemiol 1998;148:384-9.
3. Raviglione MC, Dye C, Schmidt S, Kochi A. Assessment of worldwide tuberculosis control. Lancet 1997;350:624-9.
4. Pablos-Mendez A, Raviglione MC, Laszlo A, Binkin N, Rieder HL, Bustreo F, et al. Global surveillance for antituberculosis-drug resistance, 1994-1997. N Engl J Med 1998;338:1641-9.
5. Edlin BR, Tokars JI, Grieco MH, Crawford JT, Williams J, Sordillo EM, et al. An outbreak of multidrug resistant tuberculosis among hospitalized patients with the acquired immunodeficiency syndrome. N Engl J Med 1992; 326:1514-21.
6. Beck-Sague C, Dooley SW, Hutton MD, Otten J, Breeden A, Crawford JT, et al. Hospital outbreak of multidrug resistant *Mycobacterium tuberculosis* infections. JAMA 1992;268:1280-6.
7. Kritski AL, Marques MJ, Rabahi MF, Vieira MA, Werneck-Barroso E, Carvalho CE, et al. Transmission of tuberculosis to close contacts of patients with multidrug-resistant tuberculosis. Am J Respir Crit Care Med 1996;153:331-5.
8. Welch DF, Guruswamy AP, Sides SJ, Shaw CH, Gilchrist MJR. Timely culture for *Mycobacteria* which utilizes a microcolony method. J Clin Microbiol 1993;31:2178-84.
9. Kent BD, Kubica GP. Public health mycobacteriology: A guide for the level III laboratory. Atlanta: Department of Health and Human Services, Centers for Disease Control, 1985;36-9, 47-69,185-7.
10. Franzblau SG, Witzig RS, McLaughlin JC, Torres P, Madico G, Hernandez A, et al. Rapid, low-technology MIC determination with clinical *Mycobacterium tuberculosis* isolates by using the microplate Alamar Blue assay. J Clin Microbiol 1998;36:362-6.
11. Stricof RL, DiFerdinando GT, Osten WM, Novick LF. Tuberculosis control in New York City hospitals. Am J Infect Control 1998;26:270-6.
12. Bangsberg DR, Crowley K, Moss A, Dobkin JF, McGregor C, Neu HC. Reduction in tuberculin skin-test conversions among medical house staff associated with improved tuberculosis infection control practices. Infect Control Hosp Epidemiol 1997;18:566-70.
13. Hoyos C, Izquierdo G, Piscoya G, Romero M, Saldías J. [Incidence of infective diseases at an internal medicine service]. Rev Gastroenterol Peru 1991;11:171-5.
14. Centers for Disease Control and Prevention. Multi-drug-resistant tuberculosis outbreak on an HIV ward – Madrid, Spain, 1991-1995. MMWR Morb Mortal Wkly Rep 1996;45:330-3.
15. Enarson DA, Grosset J, Mwinga A, Hershfield ES, O'Brien R, Cole S, et al. The challenge of tuberculosis: statement on global control and prevention. Lancet 1995;346:809-19.
16. Caviedes L, Lee TS, Gilman RH, Sheen P, Speelman E, Lee EH, et al. Rapid, efficient detection and drug susceptibility testing of *Mycobacterium tuberculosis* in sputum by microscopic observation of broth cultures. J Clin Microbiol 2000;38:1203-8.
